# Magnitude of change with antidepressants and placebo in antidepressant clinical trials using structured, taped and appraised rater interviews (SIGMA-RAPS) compared to trials using traditional semi-structured interviews

**DOI:** 10.1007/s00213-014-3584-4

**Published:** 2014-04-26

**Authors:** Arif Khan, James Faucett, Walter A. Brown

**Affiliations:** 1 Northwest Clinical Research Center, 1951 152nd Pl. NE Suite #200, Bellevue, WA 98007 USA; 2 Department of Psychiatry and Behavioral Science, Duke University School of Medicine, Durham, NC USA; 3 Department of Psychiatry and Human Behavior, Brown University, Box G-A1, Providence, RI 02912 USA

**Keywords:** Antidepressant clinical trials, Psychiatric interview methods, Psychological evaluation, SIGMA, MADRS, Levomilnacipran, Vilazodone

## Abstract

**Rationale:**

Although newer interview methods such as Structured Interview Guide for the Montgomery-Asberg Depression Rating Scale (SIGMA; MADRS) with audiotaping and Rater Applied Performance Scale (RAPS) appraisal have been introduced to improve reliability of ratings in antidepressant clinical trials, there is limited evidence that these methods actually improve trial outcome.

**Objective:**

The objective of this study uis to evaluate outcome in four similarly designed trials of two recently approved antidepressants: two trials randomly used taped SIGMA interviews with RAPS appraisal and two trials used traditional semi-structured MADRS interviews.

**Methods:**

We reviewed data from patients who were screened (*N* = 243) and randomized (*N* = 148), evaluating the magnitude of change with placebo and antidepressants on mean total MADRS score.

**Results:**

Depressed patients assigned to placebo in trials using taped SIGMA interviews with RAPS appraisal had a significantly larger MADRS change score (*M* = −11.5 ± 12.7) compared to patients assigned to placebo in trials using traditional semi-structured interviews (−5.4 ± 8.9; *F*(*df* = 1.57) = 5.58, *p* = 0.022). The error variance was also significantly larger in the placebo arm of trials using SIGMA interviews (*F* = 5.43, *p* = 0.023). Depressed patients assigned to antidepressants had similar outcome in all of the four trials.

**Conclusion:**

The recently suggested modifications in obtaining clinical data in antidepressant trials such as taped SIGMA interviews with RAPS rating appraisals may in fact result in a higher magnitude of placebo response and a lower magnitude of antidepressant-placebo differences compared to the traditional methods of collecting clinical data. These results were unexpected and indicate the necessity to test new methods prospectively, no matter how intuitively sensible they seem, prior to their implementation.

## Introduction

It has been suggested that the variability and unpredictability of response to antidepressants (25 to 60 % symptom reduction) and placebo (10 to 42 % symptom reduction) in antidepressant clinical trials (Khan et al. 2003) is related at least in part to poor rating interview techniques. Specifically, it is suggested that the traditional interview format is too loose and not structured enough to obtain data that are valid and similar across the different raters involved in a clinical trial (Kobak et al. 2005, 2007; Engelhardt et al. 2006).

Furthermore, it is suggested that the interview technique of a single rater may vary from interview to interview throughout a trial. Kobak et al. (2007) and Engelhardt et al. (2006) suggest that raters in antidepressant clinical trials are therefore prone to low levels of reliability. Also, there may be rater bias of inflating the entry scores to make depressed patients eligible to enter into a trial.

The idea that an increase in the reliability of ratings may enhance antidepressant-placebo differences (Lipsitz et al. 2004) was supported in a retrospective study by Cogger (2007). He evaluated the quality of a subsample of taped interviews from a paroxetine trial and noted that the effect size between antidepressant and placebo was much larger if the interviewers adhered to a specific format, asked the same questions, and took adequate time to get the best answers.

Based in part on the concept that the reliability of ratings in antidepressant trials could affect outcome, a few modifications in the conduct of recent antidepressant clinical trials were suggested: “rating the raters” (Engelhardt et al. 2006), Rater Applied Performance Scale (RAPS) appraisal (Lipsitz et al. 2004), and a Structured Interview Guide for the Montgomery-Asberg Depression Rating Scale (SIGMA; MADRS) (Williams and Kobak 2008; Montgomery and Asberg 1979). Audiotaping or videotaping interviews that are conducted either remotely (central raters) or at the investigative site (local raters) have been used to verify adherence to the modified interview formats.

Kobak et al. (2010) reported that the “central raters” trained in these structured interview techniques noted a lower magnitude of placebo response compared to the “local raters” who conducted interviews in person at the trial site. The authors attributed this difference in outcome to more reliable ratings by the “central raters.” However, three recent reports have challenged the concept that rating interview modifications have the intended effect of increasing antidepressant-placebo differences.

Oren et al. (2008) reported results from a trial of an investigational medication versus escitalopram and placebo using centralized raters who determined patient eligibility and assessed the primary outcome measure. The trial reported by Oren et al. used raters who were not based at the study site, who remotely interviewed patients via a video screen. The remote raters were unaware of the stage of the study the patient was in and the raters changed for each visit. These modifications were done to achieve uniformity of ratings, enhance reliability of ratings, and minimize rater bias. The authors reported no significant difference in outcome between placebo and escitalopram, and the results for the investigational medication were significantly worse than for placebo (*p* = 0.04).

More recently, Targum et al. (2013) reported results from a study evaluating trial outcome differences based on the method of rating: “central raters” that conducted interviews via a videoconference interview versus local site raters. A significant difference indicating superiority of combination (buspirone plus melatonin) treatment versus placebo was reported on the IDSc30 (Rush 2000) for the site-based ratings (*p* = 0.030), whereas the centralized ratings did not detect this difference (*p* = 0.124) (details reported in Table 3 of the Targum et al. publication).

Lastly, Khan et al. (2014) evaluated outcome of three clinical trials that used SIGMA interview ratings that were audiotaped and were either conducted or appraised remotely versus four clinical trials that used traditional semi-structured MADRS interviews. Our primary focus was on the outcome with the placebo groups because not all of the agents were approved for the treatment of depression by the US Food and Drug Administration (FDA). We found a larger magnitude of change in the placebo group among trials that used taped SIGMA interviews with remote scoring or appraisal, and we also found significantly more variability in the outcome with placebo in the subset of trials using these newer interview methods.

Given this background, we undertook the current study to further assess the effect of rating interview format on clinical trial results. We systematically reviewed data from the antidepressant and placebo arms of four of the antidepressant clinical trials that were included in our previous study. All of the trials used antidepressants that have been approved by the US Food and Drug Administration.

Most importantly, the trials were all conducted by the same pharmaceutical company and all used similar clinical trial design. The primary difference in the conduct of the trials was that two of the four trials used SIGMA interviews that were taped to obtain MADRS ratings and the ratings from these trials were subject to outside RAPS appraisal, whereas the other two trials used traditional semi-structured MADRS interviews. The choice of the evaluation method was randomly chosen by the sponsoring company. Our evaluation focused on the outcome in the antidepressant and placebo arms in each subset of trials.

We hypothesized that the antidepressants trials that used the newer patient evaluation techniques would result in a lower magnitude of differences between antidepressants and placebo compared to the traditional patient interview techniques. Furthermore, we hypothesized that such a difference would be related to the magnitude of change among depressed patients assigned to placebo.

## Methods

### Background

Of the 12 antidepressant trials conducted at the Northwest Clinical Research Center (NWCRC) between 2009 and 2011, only four were suitable for the current study. The other eight trials included investigational antidepressants that have not been approved or enrolled depressed patients who were treatment-resistant. The other trials conducted at NWCRC during this time were therefore not suitable for inclusion in the study.

All of the four included trials were sponsored by the same pharmaceutical company with nearly identical study design aside from the interview method. For two of the trials, the sponsoring company randomly chose to use interview modifications including site-based SIGMA interviews that were audiotaped and appraised for RAPS adherence by outside interview monitors. The other two trials were conducted using the traditional method of interviews to obtain data for MADRS scores. The NWCRC had no input as to which method was used by the company.

Although the subset of trials that used taped SIGMA interviews with outside RAPS appraisal used newer rating methods, the site-based raters that conducted the ratings were the same for each subset of trials. Each of the site-based raters had more than 2 years of experience participating in clinical trials, and the raters each had a Master’s Degree in Clinical Psychology or related field. All of the site raters also completed the study-specific rater training for each study in which they participated.

Of the four trials included in our study, three of the trials evaluated the recently approved agent levomilnacipran and one of the trials was a placebo-controlled phase IV study of vilazodone which was approved in 2011. The SIGMA interviews that were taped with outside RAPS appraisal were used during two of the levomilnacipran trials, and traditional semi-structured MADRS rating methods were used in the third levomilnacipran trial and the vilazodone trial.

The primary outcome variable for each of the trials was the change in MADRS score from baseline to final visit based on the depression scale scores of the site-based raters. The central appraisal was done post hoc and the appraisals were issued shortly after the site-based ratings were completed to encourage conformity with SIGMA style throughout the trials.

### Characteristics of the trials and demographics of the patients

The fact that clinical trial design characteristics are associated with clinical trial outcome is well documented. Factors such as the baseline severity of depression (Joyce and Paykel 1989; Khan et al. 2002) are associated with the trial outcome and may influence the proportion of patients that qualify for a trial. A lower probability of being assigned to placebo may increase patient expectations for improvement, resulting in a higher placebo response (Papakostas and Fava 2009; Rutherford et al. 2009; Sinyor et al. 2010).

We therefore critically evaluated the trial characteristics and patient demographics of the trials using the taped SIGMA interviews with RAPS appraisals as compared to the trials using the traditional semi-structured MADRS interviews prior to comparing outcome of the trials. Each of the trials recruited and enrolled patients diagnosed with major depressive disorder. Patients diagnosed with treatment-resistant depression defined as a failure of two adequate antidepressant treatment trials during their lifetime were excluded from each of the four trials.

All four trials used a randomized, parallel group, double-blind, placebo-controlled design. The visitation schedule was also identical among the four trial including a pre-randomization (“baseline”) visit as well as visits at weeks 1, 2, 4, and 6 with the final MADRS rating being assessed at the week 8 visit. As shown in Table 1, all of the trials were of 8 weeks duration. A similar number of patients were screened for study enrollment in trials using the different rating methods (taped SIGMA interview trials with RAPS appraisal, *N* = 120; traditional semi-structured MADRS interview trials, *N* = 123). Of the patients that presented for screening, there were no significant differences in age, sex, or racial characteristics between the two groups of trials.

However, there were some significant differences in the two sets of trials as described below. The two trials using the SIGMA interviews that were audiotaped and appraised had a higher entry criterion for pre-randomization severity of depression symptoms at the time of randomization. These two trials required the total MADRS score to be 30 or higher, whereas the two trials using the traditional interview techniques required the total MADRS score to be 26 or higher.

The screen fail rate of 48 % was significantly higher in trials using taped SIGMA interviews with outside RAPS appraisal compared to the screen fail rate of 31 % in the trials using traditional semi-structured interviews (*p* < 0.001). This resulted in fewer patients being enrolled in the trials using the newer interview methods (*N* = 63) compared to the trials using the traditional interview methods (*N* = 85).

The higher pre-randomization score requirement was also likely related to the higher MADRS scores of patients enrolled in the SIGMA trials (*M* = 37 ± 4) as compared to those enrolled in the trials using traditional semi-structured MADRS interview methods (*M* = 32 ± 3; *p* = 0.008). Lastly, the placebo risk was not significantly different in the two types of study as shown in Table 1. Of the patients enrolled in the trials using SIGMA interviews, 22 were assigned to placebo and 41 were assigned to antidepressants (35 % chance of receiving placebo). Of the trials conducted using traditional interviews, 37 were assigned to placebo and 48 were assigned to antidepressants (44 % chance of receiving placebo).

**Table 1:** Trial characteristics and patient demographics of those screened and enrolled in clinical trials of recently approved antidepressants levomilnacipran (trials 1, 2, and 4) and vilazodone (trial 3) based on patient rating method

	Trials using SIGMA interviews with tape recording RAPS appraisal			Trials using traditional semi-structured depression interviews			Probability^a^
	Trial 1	Trial 2	Totals (trials 1 and 2 )	Trial 3	Trial 4	Totals (trials 3 and 4)	
Trial duration	8	8	–	8	8	–	–
No. of patients screened	79	41	120	79	44	123	–
Mean age (±SD)	39 (12)	39 (14)	39 (13)	38 (12)	41 (14)	39 (13)	0.95
Female (%)	49	56	52	47	52	49	0.65
Caucasian (%)	68	81	73	70	64	68	0.39
No. of patients randomized	39	24	63	53	32	85	–
Screen fail rate	51	41	***48***	32	27	***31***	***0.008***
Mean pre-randomization score (±SD) (MADRS)	37 (5)	36 (4)	***37 (4)***	32 (3)	32 (3)	***32 (3)***	***<0.001***
Placebo exposure risk	50 %	25 %	35 %	50 %	33 %	44 %	0.29
Early discontinuation (%)	26 %	13 %	21 %	17 %	31 %	22 %	0.80

Data presented are from a single research center

Bolded italicized values indicate a significant difference between trials using the SIGMA interviews as compared to the trials using traditional non-SIGMA MADRS interviews such that the screen fail was significantly higher in trials 1 and 2 with the mean pre-randomization score also being significantly higher in trials 1 and 2.

*SIGMA* Structured Interview Guide for the Montgomery-Asberg Depression Rating Scale, *RAPS* Rater Applied Performance Scale, *MADRS* Montgomery-Asberg Depression Rating Scale

^a^Probability values were determined by chi-square test of independent samples *t* test

Aside from the higher baseline severity of depression in the trials using the SIGMA interviews with audiotaping and outside RAPS appraisal, there was very little difference in either the patient population or the design and conduct of the trials. We therefore considered it appropriate to control for the baseline severity of depression by entering it as a covariate in all of our analyses. This allowed for us to primarily analyze any difference in outcome that may have been associated with rating methodology as this design feature was the primary difference in conduct of the trials and was the focus of our evaluation.

### Analysis of data

All of the data for analysis was from one site (NWCRC) only. The results of the overall studies are being presented and published by the sponsoring pharmaceutical company.

Our first analysis was an ANOVA that evaluated the outcome for patients assigned to placebo in the trials using the SIGMA ratings that were taped with outside RAPS appraisal versus the trials that used traditional semi-structured MADRS interviews. We entered the last observation carried forward (LOCF) MADRS score as the dependent variable with the pre-randomization severity of depression as a covariate. The style of rating used to conduct the interview was the independent variable.

The next analysis focused on the outcome for patients assigned to antidepressants who were enrolled in the trials using taped SIGMA ratings with outside RAPS appraisal compared to outcome for patients assigned to antidepressants who were enrolled in the trials using traditional MADRS interviews. We again used an ANOVA with the last observation carried forward entered as the dependent variable and again controlled for the pre-randomization severity of depression by entering it as a covariate.

For each analysis, we evaluated the equality of error variance in the two subsets of trials using Levene’s test of equality of error variances. All of the analyses were conducted using SPSS versions 19.0.

Lastly, we evaluated the overall outcome with antidepressant versus placebo in each subset of trials. We again used two ANOVAs for this analysis: the first was for the SIGMA trials with taping and outside appraisal with the second ANOVA evaluating the trials using traditional, semi-structured interviews. The dependent variable was the LOCF MADRS observation with the patient assignment to antidepressants or placebo being the independent variable. We again controlled for the baseline severity of depression by entering it as a covariate in both analyses.

## Results

The mean MADRS scores at each study visit among patients included at NWCRC and assigned to placebo and antidepressants based on the rating method used in the trials are shown as Table 2. The patients that were assigned to placebo participating in trials using taped SIGMA interviews with outside RAPS appraisal had a significantly higher magnitude of placebo response (MADRS change score of −11.5 ± 12.7) compared to the patients assigned to placebo that participated in the trials using traditional semi-structured interviews (MADRS change score of −5.4 ± 8.9; *F*(*df* = 1.57) = 5.58, *p* = 0.022). There was also significantly greater error variance in LOCF evaluations of patients assigned to placebo in the trials using SIGMA methods (*F* = 5.43, *p* = 0.023).

**Table 2:** Change over time with antidepressants and placebo in two trial using SIGMA interviews with taping and outside RAPS appraisal (trials 1 and 2) compared to two similarly conducted trials using traditional semi-structured MADRS interviews (trials 3 and 4).

	Placebo		Antidepressant	
Visit	SIGMA ratings (trials 1 and 2, *N* = 20)	Traditional ratings (trials 3 and 4, *N* = 34)	Taped SIGMA ratings (trials 1 and 2, *N* = 32)	Traditional ratings (trials 3 and 4, *N* = 40)
Baseline	36.9 ± 4.8	31.8 ± 3.7	36.6 ± 4.3	31.0 ± 3.0
Week 1	32.3 ± 6.8	30.7 ± 4.4	32.1 ± 4.5	28.7 ± 4.5
Week 2	30.5 ± 8.1	29.2 ± 5.0	28.5 ± 6.1	25.2 ± 6.4
Week 4	25.7 ± 9.6	26.7 ± 8.0	23.1 ± 8.0	22.1 ± 8.0
Week 6	23.3 ± 11.9	25.9 ± 9.9	21.8 ± 10.1	16.9 ± 9.0
LOCF	*23.5* ± *13.4* ^a^	*26.0* ± *10.5* ^a^	*21.1* ± *9.3*	*15.0* ± *9.3*
Change score	−*11.5* ± *12.7* ^b^	−*5.4* ± *8.9* ^b^	−13.5 ± 9.4	−13.7 ± 10.3

*SIGMA* Structured Interview Guide for the Montgomery-Asberg Depression Rating Scale, *RAPS* Rater Applied Performance Scale, *LOCF* Last Observation Carried Forward

^a^After controlling for baseline severity of symptoms, there was a significant difference in LOCF score in the placebo arm (*F*(*df* = 1.57) = 5.58, *p* = 0.022) with the taped SIGMA trials with outside appraisal having significantly greater change score with placebo compared to the trials using traditional non-SIGMA ratings. There was also significantly more error variance at the LOCF visit in trials using SIGMA ratings (*F*(1.57) = 5.43, *p* = 0.023).

^b^The placebo change score was significantly greater in the trials using SIGMA ratings, *t*(df = 57) = 2.2, *p* = 0.034 and the variance with placebo was significantly greater in trials using SIGMA ratings, *F* = 5.5, *p* = 0.02.

There were no significant differences in change score in the antidepressant arm of the trials. The MADRS change score in the patients assigned to antidepressants in the trials using taped SIGMA interviews with outside RAPS appraisal was −13.5 ± 9.4 compared to the antidepressant change score of patients participating in the trials using traditional semi-structured interviews of −13.7 ± 10.3. There was also no significant difference in error variance in the antidepressant arms of the trials using SIGMA interviews with outside RAPS appraisal as compared to the trials using traditional interviews (*F* = 2.41, *p* = 0.12).

**Fig. 1:**
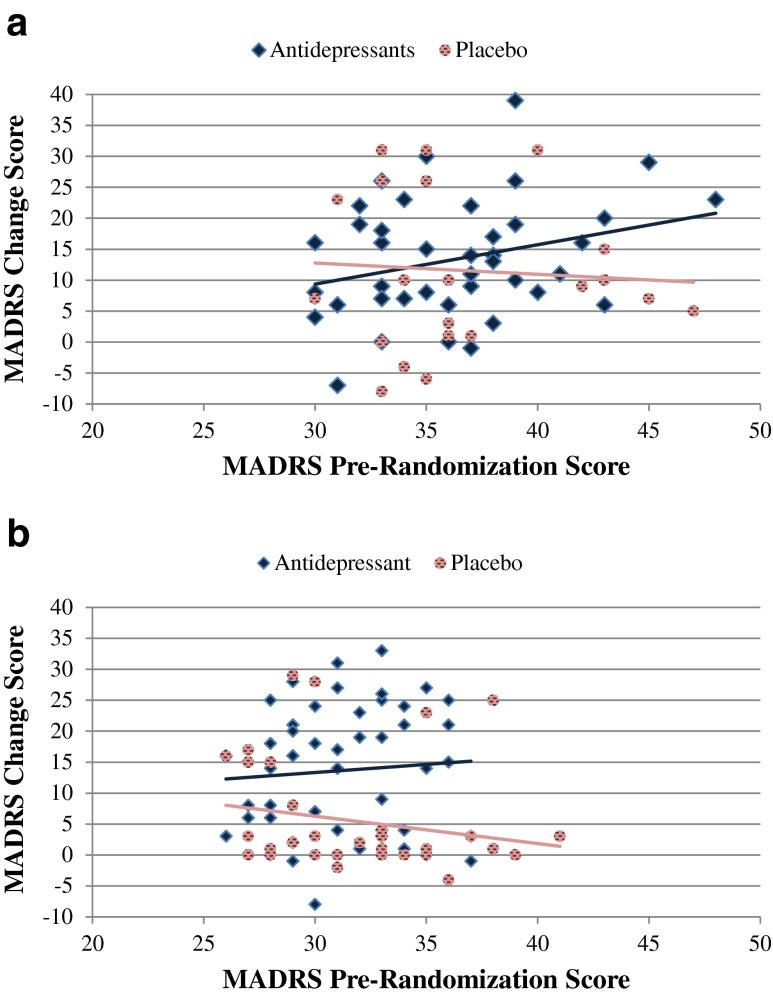
**a** Scatter plot of MADRS baseline and pre-randomization scores in trials using SIGMA interviews that are taped with RAPS appraisal from outside reviewers. There was no significant difference in outcome for patients assigned to antidepressant versus placebo (*F*(*df* = 1,61) = 0.5, *p* = 0.48). **b** Scatter plot of MADRS baseline and pre-randomization scores in trials using traditional non-SIGMA MADRS interviews. The MADRS change score was significantly higher for patients assigned to antidepressants versus placebo (*F*(*df* = 1.83) = 14.48, *p* < 0.001). *SIGMA* Structured Interview Guide for the Montgomery-Asberg Depression Rating Scale, *RAPS* Rater Applied Performance Scale, *MADRS* Montgomery-Asberg Depression Rating Scale

The comparisons of antidepressant and placebo within the subsets of trials are shown as Fig. 1a, b. Figure 1a is a scatter plot showing the relationship between MADRS pre-randomization score and MADRS change score with antidepressants and placebo in the trials using SIGMA interviews that were taped with outside appraisal. After controlling for pre-randomization severity of depression, there was not a significant difference in MADRS change score for antidepressants (−13.5 ± 9.4) compared to placebo (−11.5 ± 11.5; *F*(*df* = 1.61) = 0.504, *p* = 0.481). In these trials, the placebo arm had a larger error variance compared to the drug arm (*F* = 5.34, *p* = 0.024).

Figure 1b is a scatter plot showing the relationship between MADRS pre-randomization score and MADRS change score with antidepressants and placebo in the trials using traditional semi-structured interviews. The MADRS change score with antidepressants (−13.7 ± 10.3) was significantly larger than the MADRS change score with placebo (5.4 ± 8.9; *F*(*df* = 1.83) = 14.5, *p* < 0.001). The error variance tended to be larger in the antidepressant group (*F* = 3.7, *p* = 0.058).

## Discussion

The aim of this study was to evaluate the outcome among patients assigned to antidepressants and placebo in a group of four similarly conducted antidepressant clinical trials of two recently approved antidepressant medications; two of the trials randomly used the newer structured SIGMA format followed by a RAPS appraisal from independent reviewers with two trials using traditional semi-structured MADRS interviews. All of the analyses included only data from depressed patients who participated at a single site (NWCRC) as part of the four large multicenter trials.

Our finding supported our hypothesis that the antidepressants using the newer interview techniques would have a lower magnitude of antidepressant-placebo differences, largely due to a larger magnitude of change with placebo rather than magnitude of change with antidepressants. This finding further supports our earlier finding (Khan et al. 2014). Our study is the first to evaluate how the structured interview methods with taping of the patients and outside appraisal of the clinical trial raters may actually impact outcome of pivotal antidepressant trials as the only antidepressants that were evaluated in our study have been shown to be effective in multiple antidepressant clinical trials that have undergone FDA review.

These results are consistent with the results of the Oren et al. (2008) trial and those of Targum et al. (2013) who both reported negative findings in trials using some of the recently introduced methods of rating in antidepressant clinical trials. Oren et al. found that central raters were unable to distinguish antidepressants from escitalopram in a trial of an investigational antidepressant that has yet to be approved, whereas Targum et al. reported that the site raters were able to detect a difference in combination therapy versus placebo that the centrally based raters could not detect.

On the other hand, our findings do not support the findings of Cogger (2007) or Kobak et al. (2010) among the published trials that have used modified interview techniques of different varieties to increase the reliability of depression ratings. In other words, although it is implied that greater reliability and homogeneity of ratings will result in a better outcome, we found that newer techniques resulted in a larger and more variable placebo response in pivotal approval trials that were part of drug development programs.

In this context, it is important to note that in the past 5 years far more trials than the four included in our study have used various modifications to depression interview techniques. These modifications replace the traditional semi-structured MADRS interviews with methods including SIGMA, both central and local raters, or just central raters, and either audio- or videotaping. The data from the majority of these trials have not been systematically presented or published. Given this fact, the results of our study cannot be considered definitive until the data from these trials are fully available in the public domain. Also, our study does not evaluate the role of centralized ratings compared to site ratings.

In trying to understand our finding, we asked several raters and patients about their rating experiences. In describing her experience with the structured and appraised interviews, one rater said, “You are aware that your adherences to the exact language of the form as well as your assessment skills are being monitored. Of course, the interviewer begins to focus more on the details of the interview form and her own performance and attention is less available to focus on the patient. What is lost is the level of information that comes from a more global perception of the patient presentation, including non-verbal cues.”

Another rater had this to say about the SIGMA ratings with audiotaping and RAPS appraisal, “These are question and answer sessions and do not get into an in-depth exploration of the patient’s world that is unique to the individual. Also, I feel that patients feel that they are being scrutinized for good behavior and have to present themselves in the best light and are reluctant to criticize their experience. Simply put the patients become actors and seem to perform for the ratings.”

As a rule, patients were reluctant to sign up for the taped sessions and expressed preference for studies not requiring audiotaping. As one patient said, “I feel like big brother is watching and I have to behave myself”. At the end of taped sessions several patients made statements like, “the tape is off, right? Now, we can talk about anything I want, right?”

An additional incongruent fact in our study was that unlike the case in many previous studies, severity of pre-randomization depressive symptoms did not influence the outcome of the trial (Joyce and Paykel 1989; Khan et al. 2002, 2005). The antidepressant trials using the SIGMA interview technique, audiotaping, and RAPS review had higher pre-randomization scores, but a smaller magnitude of antidepressant-placebo differences.

These data raise concern about modifying standard psychiatric interview techniques and trial designs based on ideas that may have face validity, but have not been tested. This is a common practice in depression trials, and it may have led to many instances of type II errors. Specifically, if only modified interview formats had been used, it is possible that neither vilazodone nor levomilnacipran would have been approved by the US FDA. Thus, the recently suggested potpourri of antidepressant clinical trial design modifications should only be implemented if sufficient prospective data exist.

In conclusion, the recently suggested modifications in obtaining clinical data in antidepressant trials such as SIGMA interviews that are taped with ratings appraised by RAPS feedback may in fact result in a higher magnitude of placebo response and a lower magnitude of antidepressant-placebo differences compared to the traditional methods of collecting clinical data. These results were unexpected and indicate the necessity to test new methods prospectively, no matter how intuitively sensible they seem, prior to their implementation. Thus, caution is warranted in how clinicians interpret data from failed or negative antidepressant clinical trials.
